# The transcriptional regulator LysG (Rv1985c) of *Mycobacterium tuberculosis* activates *lysE* (Rv1986) in a lysine-dependent manner

**DOI:** 10.1371/journal.pone.0186505

**Published:** 2017-10-19

**Authors:** Marie Schneefeld, Tobias Busche, Robert Geffers, Jörn Kalinowski, Franz-Christoph Bange

**Affiliations:** 1 Department of Medical Microbiology and Hospital Epidemiology, Hannover Medical School, Hannover, Germany; 2 Microbial Genomics and Biotechnology, Center for Biotechnology, Bielefeld University, Bielefeld, Germany; 3 Research Group Genome Analytics, Helmholtz Center for Infection Research, Braunschweig, Germany; Beijing Institute of Microbiology and Epidemiology, CHINA

## Abstract

The *Mycobacterium tuberculosis* protein encoded by the Rv1986 gene is a target for memory T cells in patients with tuberculosis, and shows strong similarities to a lysine exporter LysE of *Corynebacterium glutamicum*. During infection, the pathogen *Mycobacterium tuberculosis* adapts its metabolism to environmental changes. In this study, we found that the expression of Rv1986 is controlled by Rv1985c. Rv1985c is located directly upstream of Rv1986 with an overlapping promoter region between both genes. Semiquantitative reverse transcription PCR using an isogenic mutant of *Mycobacterium tuberculosis* lacking Rv1985c showed that in the presence of lysine, Rv1985c protein positively upregulated the expression of Rv1986. RNA sequencing revealed the transcription start points for both transcripts and overlapping promoters. An inverted repeat in the center of the intergenic region was identified, and binding of Rv1985c protein to the intergenic region was confirmed by electrophoretic mobility shift assays. Whole transcriptome expression analysis and RNAsequencing showed downregulated transcription of *ppsBCD* in the Rv1985c-mutant compared to the wild type strain. Taken together, our findings characterize the regulatory network of Rv1985c in *Mycobacterium tuberculosis*. Due to their similarity of an orthologous gene pair in *Corynebacterium glutamicum*, we suggest to rename Rv1985c to *lysG*(^Mt^), and Rv1986 to *lysE*(^Mt^).

## Introduction

*Mycobacterium tuberculosis* (*Mt*) is the agent of tuberculosis and the most frequent bacterial killer worldwide due to a single infectious agent [1]. As response to the infection the immune system recruits mononuclear cells that directly become infected [2,3], leading to the formation of a granuloma [4]. In most cases, the granuloma prevents progressive infection, forming a dynamic balance between *Mt* and the immune system [5,6]. *Mt* possesses many strategies to adapt to the environment within the granuloma [2,7–10]. For example, specific transport systems offer the possibility to adapt to environmental changes [11].

Amino acid transporters have different functions such as uptake of nutrients or prevention of toxic, intracellular concentrations [12,13]. One example is the arginine exporter ArgO in *E*. *coli*. Its function is the export of arginine and canavanine to avoid accumulation that may cause toxic effects [13]. ArgO is under the transcriptional control of ArgP, a member of the family of LysR-type transcriptional regulators (LTTRs) in prokaryotes [13]. LTTRs are involved in diverse functions like virulence, quorum sensing, motility, stress response and amino acid transport [13–17]. LTTRs are functionally active as dimers, which are known to bind to palindromic DNA sequence [18]. A helix-turn-helix (HTH) motif at the N-terminus (20 to 90 amino acids from the N-terminus) facilitates binding to its target DNA sequence [18,19]. LysG, a well-studied LTTR of *Corynebacterium glutamicum* (*C*. *glutamicum*), is divergently transcribed from its target gene *lysE*, encoding a lysine exporter [11]. The promoter regions of *lysG* and *lysE* overlap within the intergenic region between both genes [11,12]. In *C*. *glutamicum*, regulation of *lysE* by LysG requires arginine or lysine as co-effector [12].

In *Mt*, two genes, Rv1985c and Rv1986, show protein sequence similarities to LysG and LysE of *C*. *glutamicum*, respectively. The Rv1985c protein was characterized as a DNA-binding protein targeting the A+T rich region of *oriC* of *Mt* [20]. Later the Rv1985c protein was obtained in crystalline form, and was named ArgP due to its homology to the regulator of the arginine exporter ArgO in *E*. *coli* [21]. The authors suggested that the gene product of Rv1985c is an LTTR, and proposed that the Rv1985c protein binds as a dimer to DNA [21]. The gene Rv1986 is located downstream of Rv1985c [22]. Although the function of the Rv1986 protein remains unknown, it has been shown to be regulated and to be targeted by memory T cells in human tuberculosis [23].

In this study, we demonstrate that the Rv1985c protein binds to its own and to the promoter region of Rv1986. The genomic organization of Rv1985c and Rv1986 in *Mt* is identical to the genomic organization of *lysG* and *lysE* in *C*. *glutamicum*, including an overlapping promoter region for both genes. Construction of a defined Rv1985c deletion mutant in *Mt* and gene expression analysis comparing the wild type strain and the mutant revealed that the Rv1985c protein activates transcription of Rv1986 in a lysine-dependent manner and autoregulates the expression of its own gene. Whole transcriptome expression analysis furthermore showed that apart from *lysE*(^Mt^), three further genes *ppsB*, *ppsC* and *ppsD* were under the control of Rv1985c protein, all of which belong to the cell wall metabolism. We propose to rename in *Mt* the Rv1985c protein to LysG(^Mt^), and to name the Rv1986 protein LysE(^Mt^).

## Materials and methods

### Bacteria and culture conditions

*Mt* H37Rv (ATCC 25618) was cultivated aerobically at 37°C in Middlebrook 7H9 broth or on Middlebrook 710 agar plates (Difco Laboratories, Detroit, MI) supplemented with 0.5% glycerol, 10% AS (0.5% bovine serum albumin fraction V, 140 mM NaCl), with 25 mM glucose and 0.05% Tween 80. When required, hygromycin (50 mg / l) was added to the culture media. For gene expression experiments, *Mt* was grown in minimal medium [24] containing 3.4 μM CaCl_2_, 0.35 μM ZnSO_4_, 7.3 mM KH_2_PO_4_, 2 mM MgSO_4_, 17.6 mM Na_2_HPO_4_, 190.8 μM ferric ammonium citrate, 0.05% Tween 80 supplemented with 100 mM glycerol and 5 mM asparagine, 10 mM aspartate, 2.5 mM arginine, 3.34 mM histidine, 5 mM lysine, 10 mM leucine or 10 mM ammonium as sole nitrogen source. For molecular cloning *E*. *coli* DH5α or *E*. *coli* M15 were used and cultivated aerobically at 37°C in Luria-Bertani (LB) broth or on LB agar plates with ampicillin (100 mg / l) if required. Bacterial growth was monitored by measuring the optical density at 600 nm (OD_600_) over time.

### Construction of an unmarked LysG(^Mt^) *Mt* knockout mutant and the complemented strain

The method of Pavelka and Jacobs [25] was used to generate a 355 bp deletion in *lysG*(^Mt^) by two-step homologous recombination. First, the genomic region of the gene was isolated from a genomic library of *Mt* [26] obtained by colony blot hybridization. A fragment containing *lysG*(^Mt^) was subcloned into pBluescript SK (-), digested with *Stu* I and *Mlu* I to generate a 355 bp deletion within *lysG*(^Mt^) (chromosomal location of the deletion: 2,229.278–2,229.634 bp), and further subcloned into the suicide plasmid pYUB657 [25], containing the *hyg* (hygromycin resistance) for selection and the *sacB* (sucrose sensitivity) for counter-selection. Co-integration of the plasmid was confirmed by PCR and Southern blot hybridization. Next, the bacteria were grown without selective pressure to enable a second crossing over, and counter-selected on 7H10 agar plates with 4% sucrose. The knockout mutant was confirmed by PCR (Table 1) and Southern blot hybridization (S1 Fig).

**Table 1:** The oligonucleotides used for PCR.

Primer	Sequence 5’ to 3’	Length (bp)
lysG fwd	TTCAAGCGTTTCCGTCTGAG	20
lysG rev	TTCACGCAATCGACACTAGG	20
Del fwd	TGAACCCGTCGGATAGATGG	20
Del rev	TCAGTCAGCGCATCAAGTCG	20

To obtain the complemented strain the intact gene *lysG*(^Mt^) was cloned into the integrating vector pMV306.hyg. The complemented strain was confirmed by PCR and Southern blot hybridization.

### Construction and expression of plasmid pQE30-LysG(^Mt^)x6His

To obtain a His_6_-tagged LysG(^Mt^) fusion protein the method of Gibson was used [27]. LysG(^Mt^) was amplified from chromosomal DNA of *Mt*. For the assembly of the purified PCR product with the linearized plasmid pQE30 (Qiagen, Hilden, Germany), the Gibson Assembly^™^ Cloning kit (New England Biolabs GmbH, Frankfurt am Main, Germany) was used according to the protocol given by the manufacturer.

The His_6_-tagged LysG(^Mt^) fusion protein was expressed in *E*. *coli* M15 in the presence of 1 mM IPTG (isopropyl-β-D-thiogalactopyranoside). Cells were harvested by centrifugation at 4,000 g for 20 min. The purification was performed with nickel-nitrilotriacetic acid affinity chromatography using the Ni-NTA Fast Start Kit (Qiagen, Hilden, Germany) following the instructions given by the manufacturer. The purification was analyzed by SDS-PAGE and western blot. For quantification, the Bradford method was used [28].

### Electrophoretic mobility shift assay with LysG(^Mt^)

To generate oligonucleotides II to IV, six single-strand and complementary oligonucleotides (Primer 833 to 838 in Table 2), three of which were biotin-labeled, were purchased (Eurofins Genomics GmbH, Ebersberg, Germany). Assembly of double-strand oligonucleotides were done in 10 mM Tris buffer (pH 8.0; containing 1 mM EDTA, 50 mM NaCl) using a mixture of both starnds at a 1: 1 molar ratio in a final concentration of 1 pmol /μl. Annealing temperature was initially at 95°C for 5 min, reducing stepwise to room temperature using a thermocycler (TProfessional Basic, Biometra GmbH, Göttingen, Germany). Oligonucleotides I, V to VIII (Table 2) were generated by PCR using the following primers (Eurofins Genomics GmbH, Ebersberg, Germany). The reverse primer contained a biotin-tag at the 5’-end (Primer lysE(^Mt^) rev, 834, 836, 838, fadD26 rev, ppsB rev, ppsC rev and ppsD rev):

**Table 2:** The oligonucleotides used in this study.

Oligo	Primer	Sequence 5’ to 3’	Length (bp)
I	lysE(^Mt^) fwd	CTCAGGTGTAGACCATC	17
I	lysE(^Mt^) rev	GCAATCGACACTAGG	15
II	833	CTCAGGTGTAGACCATCTGCGGAGCGTCGCACTGCACATTAATAATGCTAATGT	54
II	834	ACATTAGCATTATTAATGTGCAGTGCGACGCTCCGCAGATGGTCTACACCTGAG	54
III	835	AAATGAAGAATTATTAGCTATACTGACCCATACAAACTGCCTAGTGTCGATTGC	54
III	836	GCAATCGACACTAGGCAGTTTGTATGGGTCAGTATAGCTAATAATTCTTCATTT	54
IV	837	CGCACTGCACATTAATAATGCTAATGTAAATGAAGAATTATTAGCTATACTGAC	54
IV	838	GTCAGTATAGCTAATAATTCTTCATTTACATTAGCATTATTAATGTGCAGTGCG	54
V	fadD26 fwd	TTCAGACCGGCACGTTTCAG	20
V	fadD26 rev	ACCGGCATCGCCTTGTACTC	20
VI	ppsB fwd	ACCGCACGATGTGTCACAAG	20
VI	ppsB rev	TCGCATCACACCGACCTCTC	20
VII	ppsC fwd	GCCTGAGCTCCTCGAAATTG	20
VII	ppsC rev	CGCTGCGGTCATTGTGTTCC	20
VIII	ppsD fwd	GCGCAGGAGATTTCCGATAC	20
VIII	ppsD rev	GTTCGGCGACAGTTG	15

Labeled oligonucleotides, purified His_6_-tagged LysG(^Mt^) and 5 mM lysine or 3.34 mM histidine were mixed and incubated at room temperature for 20 min with the LightShift Chemiluminescent EMSA kit (Thermo Fisher Scientific Inc., Rockford, USA) according to the protocol given by the manufacturer. The binding reactions were analyzed on a 6% polyacrylamide gel in 0.5 x TBE buffer. For the competitive EMSA, different concentrations (pmole) of unlabeled specific competitor DNA were added.

### Extraction of RNA

RNA from bacteria was extracted from a growing culture at OD_600_ of ~0.3. The cells were incubated in an equal amount of 5 M GTC buffer (5 M guanidinium isothiocyanate, 0.5% *n*-laurylsarcosine, 0.7% sodium citrate, 0.7% β-mercaptoethanol) for 15 min at room temperature and harvested by centrifugation at 4,500 x g for 15 min. The pellet was resuspended in 1 ml of TRIzol RNA Isolation Reagent (Life Technologies, Darmstadt, Germany) and incubated for further 15 min at room temperature. Cell lysis was done in a Lysing Matrix B tube (MP Biomedicals, Illkirch, France) using the Hybaid RiboLyser. The tubes were centrifuged at 13,000 x g for 3 min and the supernatant was transferred in a new reaction tube for chloroform extraction. The aqueous solution, consisting of the RNA, was mixed with an equal volume of 70% ethanol and the purification was performed with the RNeasy Mini Kit (Qiagen, Hilden, Germany) according to the protocol given by the manufacturer. In deviation from the protocol, the optional DNase I digestion (DNase I supplied by Qiagen) was extended to 1 h and a second DNase I digestion (DNase I supplied by NEB) was performed after elution from the columns. A second RNA cleanup was done using the RNeasy Mini Kit.

### RNA sequencing

RNA quality and quantity was checked by using an Agilent 2100 Bioanalyzer (Agilent Technologies, Böblingen, Germany) and the Xpose sytem (Trinean, Gentbrugge, Belgium) prior and after rRNA depletion by using the Ribo-Zero rRNA Removal Kit (Bacteria) (Illumina, San Diego, CA, USA). The TruSeq Stranded mRNA Library Prep Kit (Illumina, San Diego, CA, USA) was used to prepare the cDNA libraries for differential transcriptome analysis. An additional library was prepared from a pool of all isolated RNA according to the protocol of Pfeifer-Sancar *et al*. [29] focusing on native 5’-ends of transcripts. The resulting cDNAs were then sequenced in paired end mode on a MiSeq system (Illumina, San Diego, CA, USA) using 2 x 75 nt read length. The raw sequencing read files are available in the ArrayExpress database (www.ebi.ac.uk/arrayexpress) [30] under Accession No.: E-MTAB-6011. Reads were mapped onto the genomic reference sequence of *Mycobacterium tuberculosis* H37Rv with Bowtie 2 [31] using standard settings. ReadXplorer 2.2.0 [32] was used for visualization of short read alignments and data analysis including transcript start site determination.

### Gene expression analysis of single genes

For transcription of 2 μg total RNA into cDNA the reverse transcriptase SuperScript II Reverse Transcriptase (Life Technologies, Darmstadt, Germany) was used following the instructions given by the manufacturer. The first-strand cDNA synthesis was performed in a T3000 Thermocycler (Biometra, Göttingen, Germany). To verify efficient DNase I treatment, for each RNA sample, two reactions were prepared, while one was treated equally except no enzyme was added (no template control). TaqMan probes were designed and ordered according to the recommendation of the manufacturer (Life Technologies, Darmstadt, Germany). Assigned sequence accession numbers were as follows (Table 3):

**Table 3:** Sequence accession number received from the manufacturer.

TaqMan probe^a^	Target gene	Sequence accession number
sigA-ANY	*sigA*	AIJ9WY5
SIGE	*sigE*	AI20T98
RV1986_MTB	Rv1986	AI20TFY

^a^Life Technologies, Darmstadt, Germany.

Samples were measured in triplicates in a 96-well plate using the C1000^™^ Thermal Cycler (Bio-Rad Laboratories GmbH, München, Germany). For quantification, a standard curve obtained by serial dilution was determined by joining all cDNAs. For normalization, the expression level of the validated reference genes *sigA* and *sigE* were used. The software CFX manager served as qPCR analysis program (Bio-Rad Laboratories GmbH, München, Germany), while the calculation method of Pfaffl [33] was used to get significant results. The cutoff for significant regulation was set at 2-fold change.

### Whole transcriptome expression analysis

For whole transcriptome expression analysis, a custom-designed Affymetrix GeneChip MTbH37Rva520456F array was used following the instructions of the manufacturer (Affymetrix, Santa Clara, USA) and as described previously [34]. The software Affymetrix GCOS 3.1 was utilized to extract raw data signals after image analysis. Raw signal intensities were log2 transformed and normalized using the Robust Multi-Array Average (RMA) algorithm. For testing differential gene expression, normalized data sets were filtered for informative genes (showing at least expression values > log2 (50) in more than two samples). For statistical analysis and assessing differential expression the R package “Limma” was used (Stratagene, La Jolla, USA). The entire data set was made publicly available under Accession No. GSE96639 at NCBI GEO database (https://www.ncbi.nlm.nih.gov/geo/). The log-log plot was generated by taking for each gene the average of all log2 data (from whole transcriptome expression analysis) from the mutant strain Δ*lysG* and plotted against those from the Wt *Mt* strain.

### Bioinformatics and structure analysis of LysE(^Mt^) and LysG(^Mt^)

The nucleotide and protein sequences were retrieved from NCBI database (http://www.ncbi.nlm.nih.gov). The multiple sequence alignment was carried out using the software SeaView version 4 [35]. The helix-turn-helix motifs were predicted via the NPSA server [36], while the hydropathy plot of proteins was obtained according to the algorithm of Kyte and Doolittle [37].

### Statistical analysis

One-way ANOVA with Tukey’s multiple comparison tests was used for the analysis of statistical significance. Statistical significance is depicted as *: p < 0.05 or **: p < 0.01.

## Results

### Genomic organization of *lysG*(^Mt^) and *lysE*(^Mt^)

**Fig 1:**
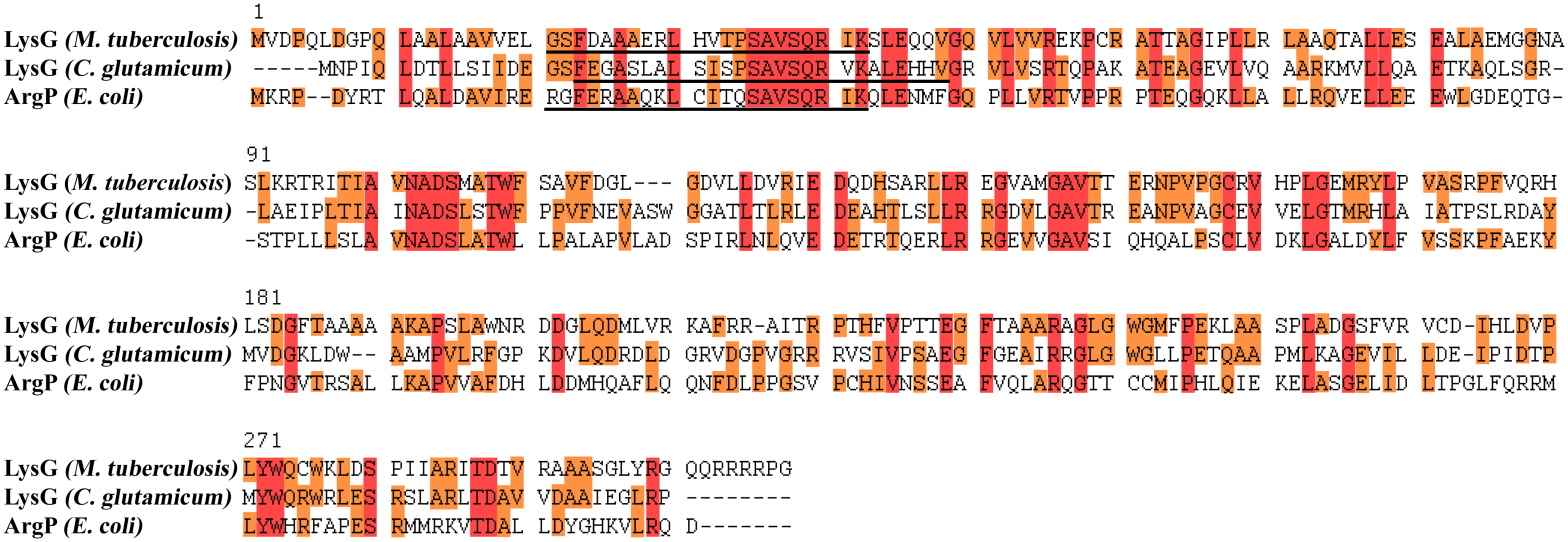
Sequence alignment revealed strong similarities between LysG(^Mt^), LysG and ArgP. Conserved amino acids are indicated in orange and fully conserved in red. The helix-turn-helix motif is underlined.

Due to the fact that the gene products of both, Rv1985c and Rv1986 [renamed in this study as LysG(^Mt^) and LysE(^Mt^)], are homologues to LysG and LysE in *C*. *glutamicum*, we were interested in studying the LysG(^Mt^) / LysE(^Mt^) regulatory unit. First, we compared the amino acid sequence of LysG(^Mt^) with other LTTRs such as ArgP of *E*. *coli* and LysG of *C*. *glutamicum*. The multiple alignment showed strong similarities between LysG(^Mt^) and the other LTTRs (Fig 1). All have an HTH motif at the N-terminus (underlined). The amino acid sequences showed a high degree of sequence similarity within the HTH motif. The HTH motif itself has the same length of 22 amino acids in ArgP and LysG(^Mt^), while the length of the HTH motif in LysG of *C*. *glutamicum* is 25 amino acids. LysG(^Mt^) showed the highest degree of homology with LysG of *C*. *glutamicum* (42% identity), and less homology with ArgP of *E*. *coli* (36% identity).

**Fig 2:**
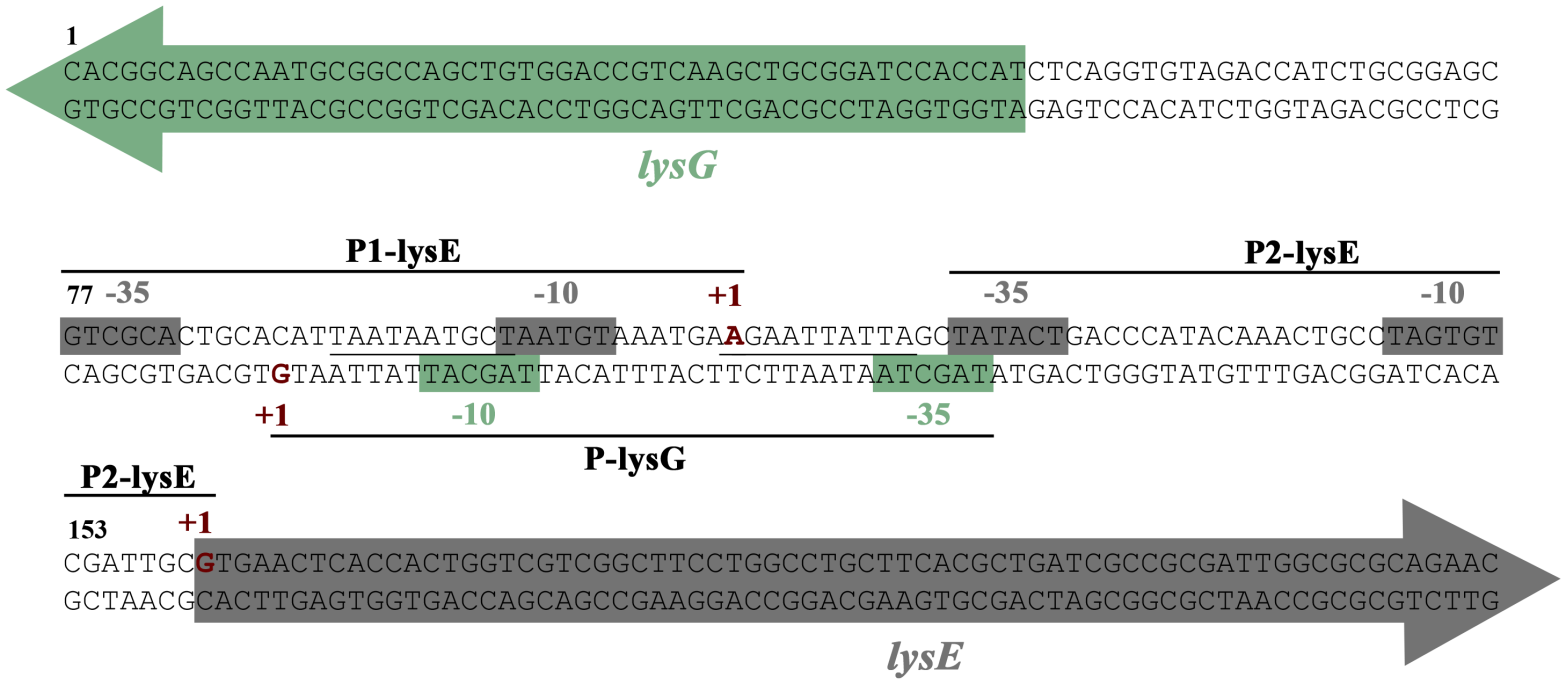
Genomic organization suggest a LTTR-dependent regulation of *lysE*(^Mt^). A 108 bp region separates *lysG*(^Mt^) from *lysE*(^Mt^). The *lysG*(^Mt^) and *lysE*(^Mt^) transcriptional start sites are indicated with +1 (in red and bold type). The coloured arrows denote the beginning of the ORFs. The promoter binding motifs (-10 and -35 motifs) are marked as boxes. The underlined base pairs represent a palindromic DNA sequence.

*LysG*(^Mt^) and *lysE*(^Mt^) are separated by a short intergenic region of 108 bp (Fig 2). By RNA sequencing of the intergenic region, we identified the transcription start sites of both genes, and found overlapping -10 and -35 promoter binding motifs for both *lysG*(^Mt^) (P-lysG) and *lysE*(^Mt^) (P1-lysE). *LysG*(^Mt^) is divergently transcribed to *lysE*(^Mt^). The center of the overlapping promoter contains an inverted repeat (palindromic DNA sequence [91 to 100: GCTAAT; 112 to 121: ATTAGC]), which might represent a potential binding region for a transcriptional regulator (Fig 2). The organization of the transcription start site and the inverted repeat suggests that expression of *lysG*(^Mt^) and *lysE*(^Mt^) is controlled by a single regulator. The intergenic region contains a second promoter for lysE (P2-lysE), the details of which are described below.

The amino acid sequence alignment of LysE(^Mt^) revealed a high degree of sequence homology with ArgO of *E*. *coli* (38% identity on the amino acid level) and LysE of *C*. *glutamicum* (32% identity on the amino acid level) (S2A Fig). Based on amino acid-homology, LysE(^Mt^) has been suggested to belong to the LysE superfamily [11]. A hydropathy plot comparing LysE(^Mt^) with that of LysE of *C*. *glutamicum* [11] indicated five transmembrane-spanning helices for LysE(^Mt^), as seen for other members of the LysE superfamily (S2B Fig). This further supported the idea that LysE(^Mt^) and LysG(^Mt^) are functionally related.

### A Δ*lysG*(^Mt^) mutant lacks upregulation of *lysE*(^Mt^) gene expression

**Fig 3:**
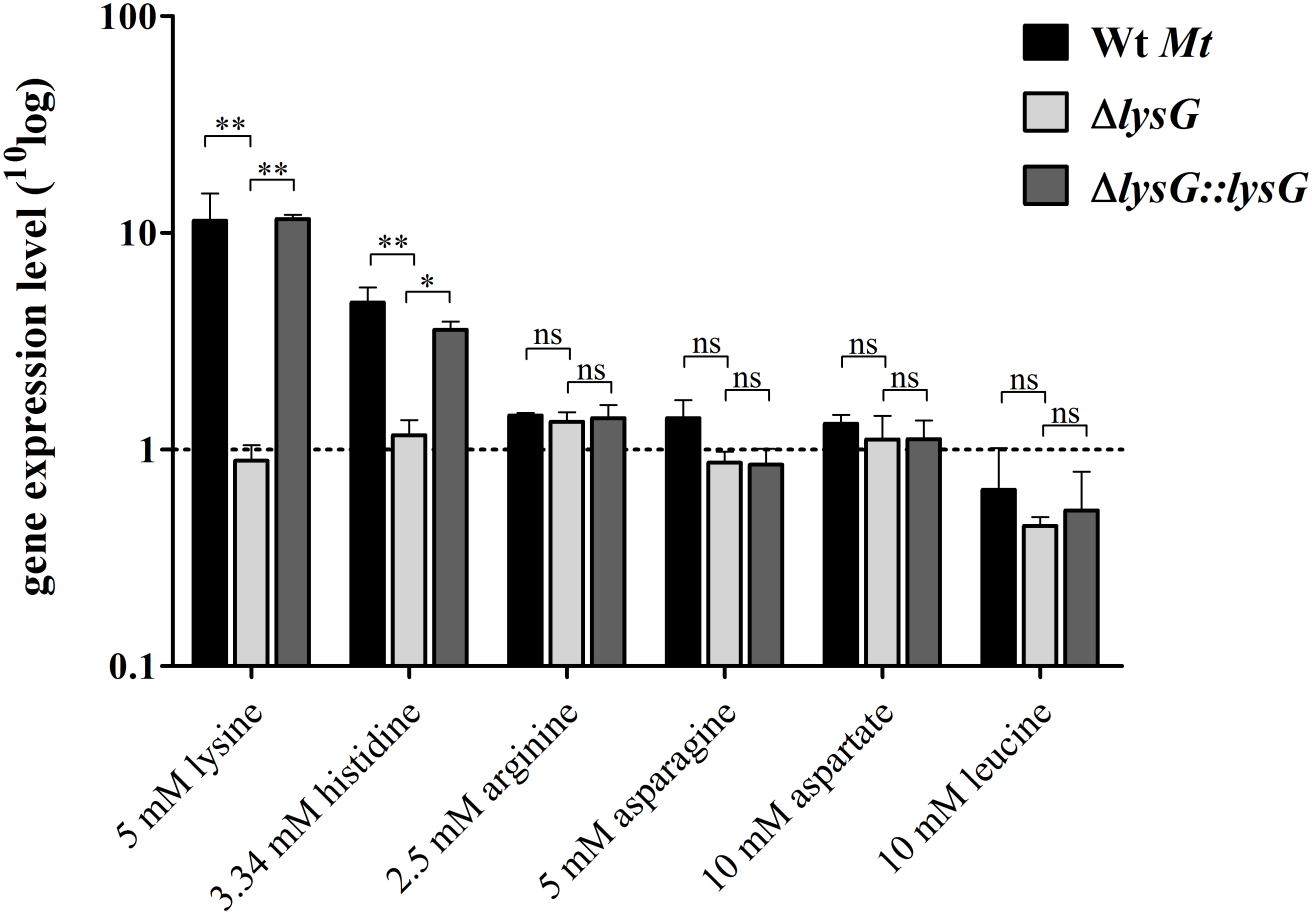
LysG(^Mt^) upregulates *lysE*(^Mt^) in the presence of lysine and histidine. Relative gene expression level of *lysE*(^Mt^) in Wt *Mt* H37Rv, the knockout mutant Δ*lysG* and the complemented strain Δ*lysG*::*lysG* during incubation in a minimal medium with lysine, histidine, arginine, asparagine, aspartate and leucine as additional nitrogen source. For normalization, the expression level of the validated reference gene *sigA* and *sigE* were used. The cutoff for significant regulation was set at 2-fold change. Means were calculated from three independent experiments. Error bars represent standard errors of the mean.

To check if LysG(^Mt^) is the transcriptional regulator of *lysE*(^Mt^), we generated an isogenic mutant of *Mt* lacking *lysG*(^Mt^). The relative gene expression level of *lysE*(^Mt^) was analyzed using semiquantitative reverse transcription PCR under different conditions. Due to the fact that most LTTRs dependent on at least one co-effector, we tested lysine, which is a co-effector for LysG in *C*. *glutamicum*, but also other amino acids including asparagine, aspartate, arginine, histidine and leucine. The wild type strain (Wt *Mt*), the knockout mutant (Δ*lysG*) and the complemented strain (Δ*lysG*::*lysG*) were cultivated in a minimal medium with different amino acids as sole nitrogen source. Our data showed a transcriptional activation of 10-fold by LysG(^Mt^) in the presence of 5 mM lysine and a 4-fold increase in gene transcription in the presence of histidine (Fig 3). The induction of gene expression was abolished in the Δ*lysG* strain. The complemented strain Δ*lysG*::*lysG* was able to restore the transcriptional activation. In contrast, asparagine, aspartate, arginine and leucine were not able to induce gene expression. This finding indicates that lysine and histidine are co-effectors of LysG(^Mt^) and thus might be involved in gene expression of *lysE*(^Mt^).

### Binding of LysG(^Mt^) to the promoter region of *lysG*(^Mt^) / *lysE*(^Mt^)

**Fig 4:**
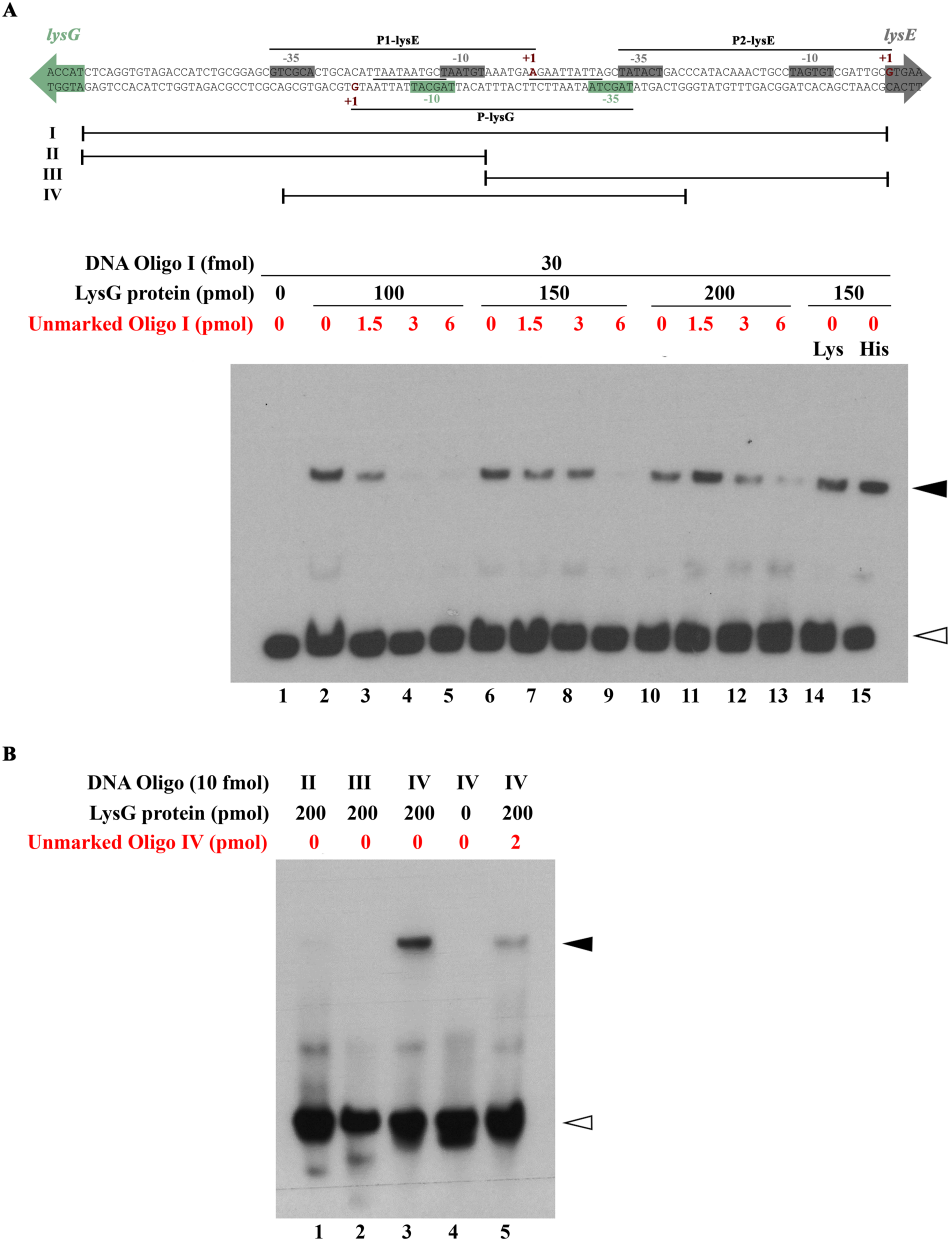
LysG(^Mt^) binds to the upstream region of *lysE*(^Mt^). (A) Intergenic region between *lysG*(^Mt^) and *lysE*(^Mt^). The *lysG*(^Mt^) and *lysE*(^Mt^) transcriptional start sites are indicated with +1 (in red and bold type). The promoter binding motifs (-10 and -35 motifs) are marked as boxes. The coloured arrows denote the beginning of the open reading frames. The location of the inverted repeat within the intergenic region is marked (underlined). The position of the labeled oligonucleotides used for the electrophoretic mobility shift assay is shown (I to IV). 30 fmole of labeled oligonucleotide I was incubated with increasing concentrations of LysG(^Mt^), indicated for each lane. As control no protein (lane 1) and 50, 100 or 200-fold molar excess of unlabeled target DNA (oligonucleotide I) were added, marked in red. 5 mM of the co-effector lysine (lane 14) and 3.34 mM of histidine were added (lane 15). Bands corresponding to free DNA are marked by open arrowheads, whereas bands with DNA in complex with LysG(^Mt^) are marked by filled arrowheads. Results are representative of two independent experiments. (B) Electrophoretic mobility shift assay with 200 pmole of LysG(^Mt^) was done in combination with 10 fmole of the three subfragments (oligonucleotides II to IV). As control no protein (lane 4) and 200-fold molar excess of unlabeled target DNA (lane 5) were added.

Next, we analyzed binding of LysG(^Mt^) to the upstream region of *lysE*(^Mt^) by electrophoretic mobility shift assay (EMSA). LysG(^Mt^) (purified His_6_-tagged protein) specifically bound to the oligonucleotide I, which comprises the whole intergenic region (Fig 4A). The use of molar excess of unlabeled specific competitor DNA (oligonucleotide I) decreased the intensity of the bound probe, indicating a specific protein-DNA-complex. In principle, the binding of the regulator could significantly change in presence of the co-effector amino acid. To determine if lysine or histidine have an influence on the binding properties of LysG(^Mt^), 5 mM of lysine or 3.34 mM histidine were added to the binding reaction. It appears that the addition of amino acids had no significant effect on the binding (Fig 4A, lane 14 and 15). The binding region for a regulator protein is often a palindromic DNA sequence. To further define the binding region of LysG(^Mt^), mainly the inverted repeat within the intergenic region, two shorter oligonucleotides (II and III) covering specific areas of the promoter region were used. The oligonucleotide II encompasses the -10 promoter binding motif of *lysG*(^Mt^) and one part of the inverted repeat, whereas the oligonucleotide III contains the other part of the inverted repeat and the second promoter (P2-lysE) of the target gene *lysE*(^Mt^). No protein-DNA-complexes were detected with the oligonucleotides II and III (Fig 4B). Thus, we tested a third oligonucleotide IV containing the entire inverted repeat, which turned out to be bound by LysG(^Mt^) (Fig 4B).

### Characterization of the *lysG*(^Mt^)-*lysE*(^Mt^) intergenic region by Transcriptome sequencing (RNAseq)

**Fig 5:**
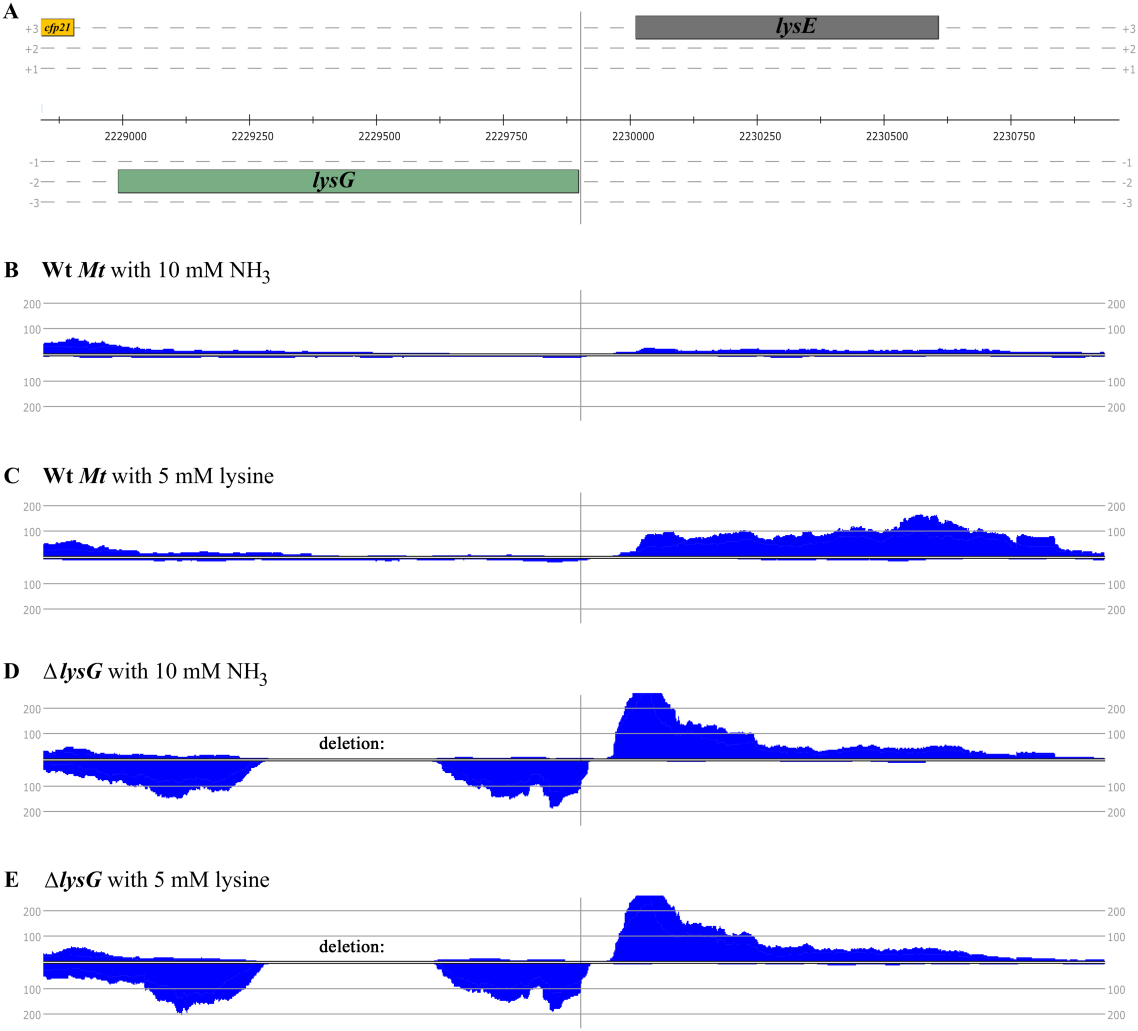
Transcriptome profiles of the DNA containing *lysG*(^Mt^) and *lysE*(^Mt^). (A) Genomic region with coding sequences. (B) and (C) Profiles of mapped reads from RNAseq using RNA samples from Wt *Mt* with ammonium (B) or lysine (C) as nitrogen source. (D) and (E) Profiles of mapped reads from RNAseq using RNA samples from the Δ*lysG* mutant strain with ammonium (D) or lysine (E) as nitrogen source.

Transcriptome sequencing was carried out with RNA samples isolated from the Wt *Mt* and the Δ*lysG* mutant strain cultivated either with ammonium or lysine supplementation. Upregulation of transcription of *lysE*(^Mt^) in presence of lysine (Fig 5) was clearly in the Wt *Mt*. Transcription was initiated form a second promoter of *lysE*(^Mt^), P2-lysE. The transcription site of this second is located at the first nucleotide of the coding region (Fig 2), leading to a leaderless transcript. This second *lysE*(^Mt^) promoter (P2-lysE) became visible only by RNAseq analysis in presence of lysine.

In the Δ*lysG* mutant, *lysG*(^Mt^) and *lysE*(^Mt^) were derepressed, and transcription of *lysG*(^Mt^) and *lysE*(^Mt^) occurred from the promoters within the inverted repeat region (P-lysG, P1-lysE). Interestingly, whereas *lysG*(^Mt^)-RNA showed a stable profile, *lysE*(^Mt^)-RNA profile showed a decrease towards the 3’-prime end.

### Whole transcriptome expression analysis identified additional genes regulated by LysG(^Mt^)

To evaluate if other genes are under the transcriptional control of LysG(^Mt^), a whole transcriptome expression analysis of Wt *Mt* and Δ*lysG* was carried out by using Affymetrix microarray technology. Both strains were grown in a minimal medium supplemented with 5 mM lysine as sole nitrogen source. Signal intensities of the mutant strain Δ*lysG* were compared to the Wt *Mt* strain (Table 4 and S1 Table). The *lysE*(^Mt^) gene expression in the Wt *Mt* was four times higher (fold change of -4.06) than in Δ*lysG*. This data confirmed the results obtained by semiquantitative reverse transcription PCR (see Fig 3). Interestingly, the gene expression level of *lysG*(^Mt^) was 8-fold upregulated in the mutant strain Δ*lysG* (Table 4), indicating that LysG(^Mt^) represses its own transcription. A signal for *lysG*(^Mt^) was detected in the mutant strain Δ*lysG*, because the deletion did not encompass the entire gene, but approximately 40% of *lysG*(^Mt^).

**Table 4:** Genes regulated by LysG(^Mt^).

Gene name		FC (abs)^a^	P-value (adj.)^b^	Gene description [22]
Rv2932	*ppsB*	-16.168	0.0000030	Phenolpthiocerol synthesis type-I polyketide synthase PpsB
Rv2933	*ppsC*	-11.793	0.0000633	Phenolpthiocerol synthesis type-I polyketide synthase PpsC
Rv2934	*ppsD*	-5.651	0.0009141	Phenolpthiocerol synthesis type-I polyketide synthase PpsD
Rv1986	*lysE*(^Mt^)	-4.056	0.0016333	Probable conserved integral membrane protein
Rv1985c	*lysG*(^Mt^)	8.482	0.0000007	Probable transcriptional regulatory protein (probably LysR-family)

Whole transcriptome expression profile of Wt *Mt* and Δ*lysG* grown in the presence of lysine. Signal intensities of the mutant strain Δ*lysG* were compared to the Wt *Mt* strain. Both were grown in a minimal medium with 5 mM lysine as sole nitrogen source.

^a^Significantly regulated genes were determined using a cutoff > 2 for an absolute fold change (FC). Fold changes were expressed as mean FC from three independent experiments.

^b^Significantly regulated genes were determined using a cutoff < 0.01 for an adjusted p-value.

**Fig 6:**
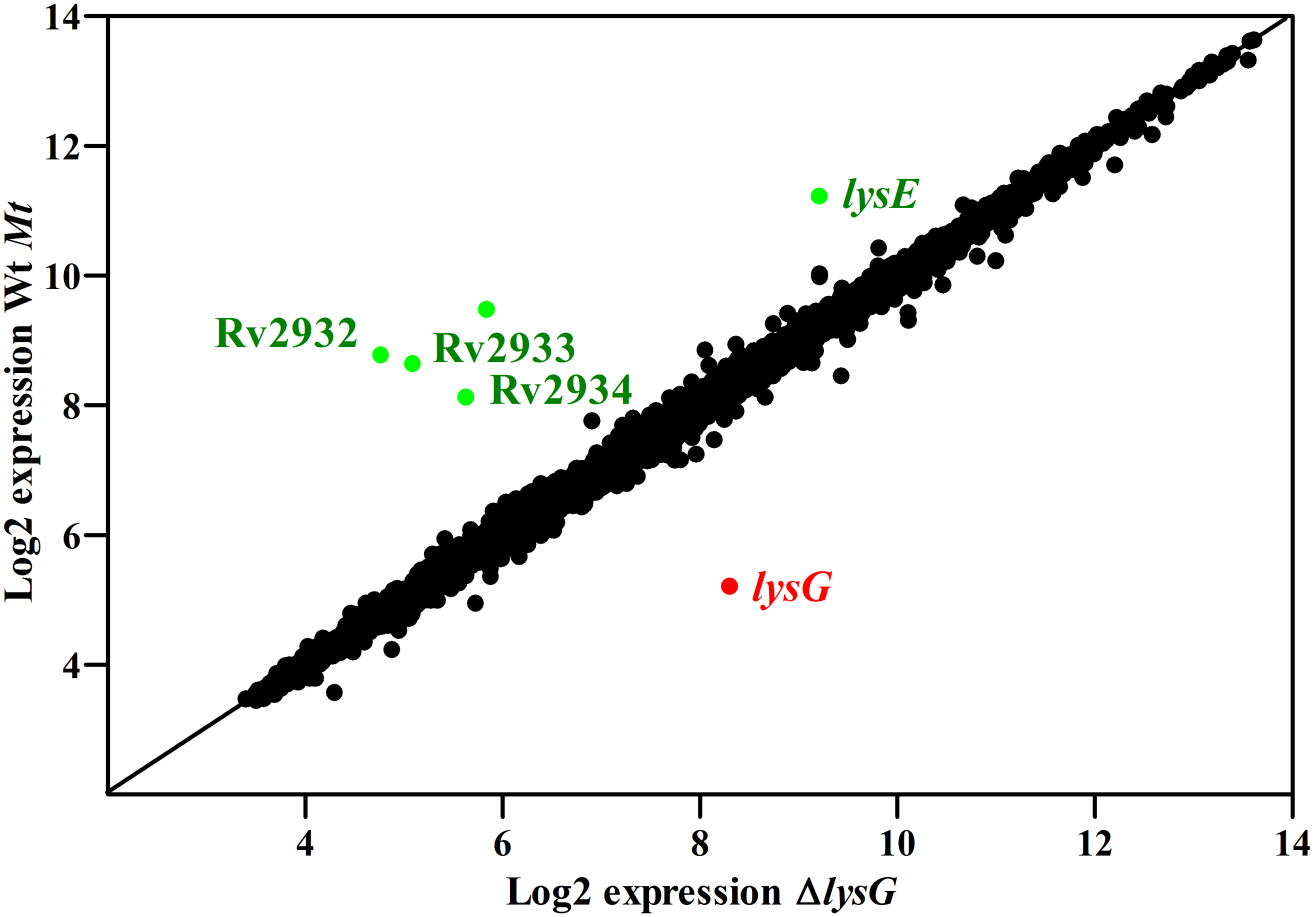
Log-log plot of the whole transcriptome expression analysis. The log-log plot shows the signal intensities of the mutant strain Δ*lysG* plotted against those of the Wt *Mt* strain. Spots represent the average of log2 data for each gene from three independent experiments. High through low expression levels are presented as a three-color spectrum. The red color indicates high expression and the green color low expression.

Three further genes, namely *ppsB*, *ppsC* and *ppsD*, were found to be downregulated (Table 4). The expression level of *ppsB* in Δ*lysG* was downregulated by factor 16 (fold change of -16.17), as well as *ppsC* which showed an 11-fold downregulation (fold change of -11.79) and *ppsD* which showed a 5-fold downregulation (fold change of -5.65). Although five genes (*ppsABCDE*) are essential for synthesis of phthiocerol [38], there was only a difference in the gene expression pattern of the genes *ppsBCD* in the knockout strain Δ*lysG* compared to the Wt *Mt*. The expression data of the Wt *Mt* and the Δ*lysG* strain are summarized in a log-log plot (Fig 6). The log-log plot presents the whole transcriptome expression analysis. Except for *ppsBCD* and for *lysEG*, the other genes showed no difference in the gene expression pattern between the Wt *Mt* and Δ*lysG* (Fig 6). These results suggest that LysG(^Mt^) functions not only as activator for gene expression of *lysE*(^Mt^) but also for the three genes *ppsBCD* and in addition autoregulates its own transcription.

### Binding of LysG(^Mt^) to the promoter region of *ppsB*, *ppsC* and *ppsD*

**Fig 7:**
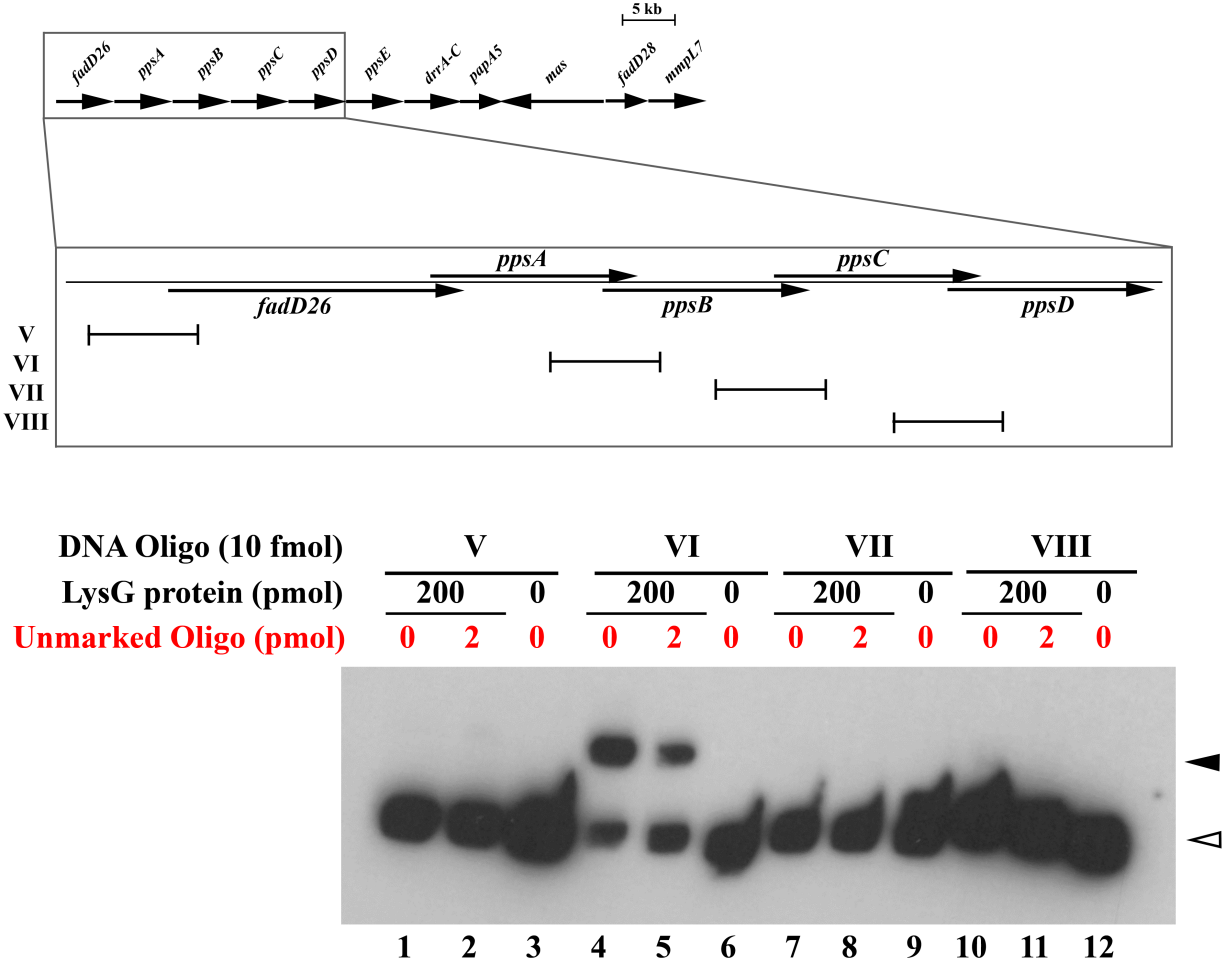
LysG(^Mt^) binds to the upstream region of *ppsB*. Genomic organization of the genes *fadD26* and *ppsABCD* of *Mt* is shown. The arrows represent the ORFs. Electrophoretic mobility shift assay with LysG(^Mt^) was done in combination with the oligonucleotides V to VIII, covering the upstream region of *fadD26* and the three genes *ppsBCD*, which were found to be regulated by LysG(^Mt^). As control no protein and 200-fold molar excess of unlabeled target DNA (oligonucleotide V to VIII) were added. Bands corresponding to free DNA are marked by open arrowheads, whereas bands of DNA in complex with LysG(^Mt^) are marked by filled arrowheads. Results are representative of two independent experiments.

To determine the binding region of LysG(^Mt^) upstream from *ppsBCD*, we incubated different oligonucleotides (V to VIII) of defined sequence with purified LysG(^Mt^) by EMSA. It is noteworthy that the genes *ppsBCD* are members of a gene cluster of 13 genes (*fadD26* to *mmpL7*), while the genes *fadD26* to *ppsD* overlap on the same strand (Fig 7). We amplified the region upstream from *fadD26*. We further amplified approximately 120 bp of the region upstream from the respective genes *ppsBCD* that are under the transcriptional control of LysG(^Mt^). LysG(^Mt^) bound to the oligonucleotide VI, representing the upstream region (117 bp) of *ppsB*, showing a protein-DNA-complex of strong intensity (Fig 7). In contrast, no protein-DNA-complex was formed with the oligonucleotides V, VII and VIII, containing the upstream region of *fadD26*, *ppsC* and *ppsD*. This corresponds to the fact, that only the genes *ppsBCD* are upregulated by LysG(^Mt^) and not *fadD26*, *ppsA* and *ppsE*. This data showed that LysG(^Mt^) binds upstream of *ppsB*.

**Fig 8:**
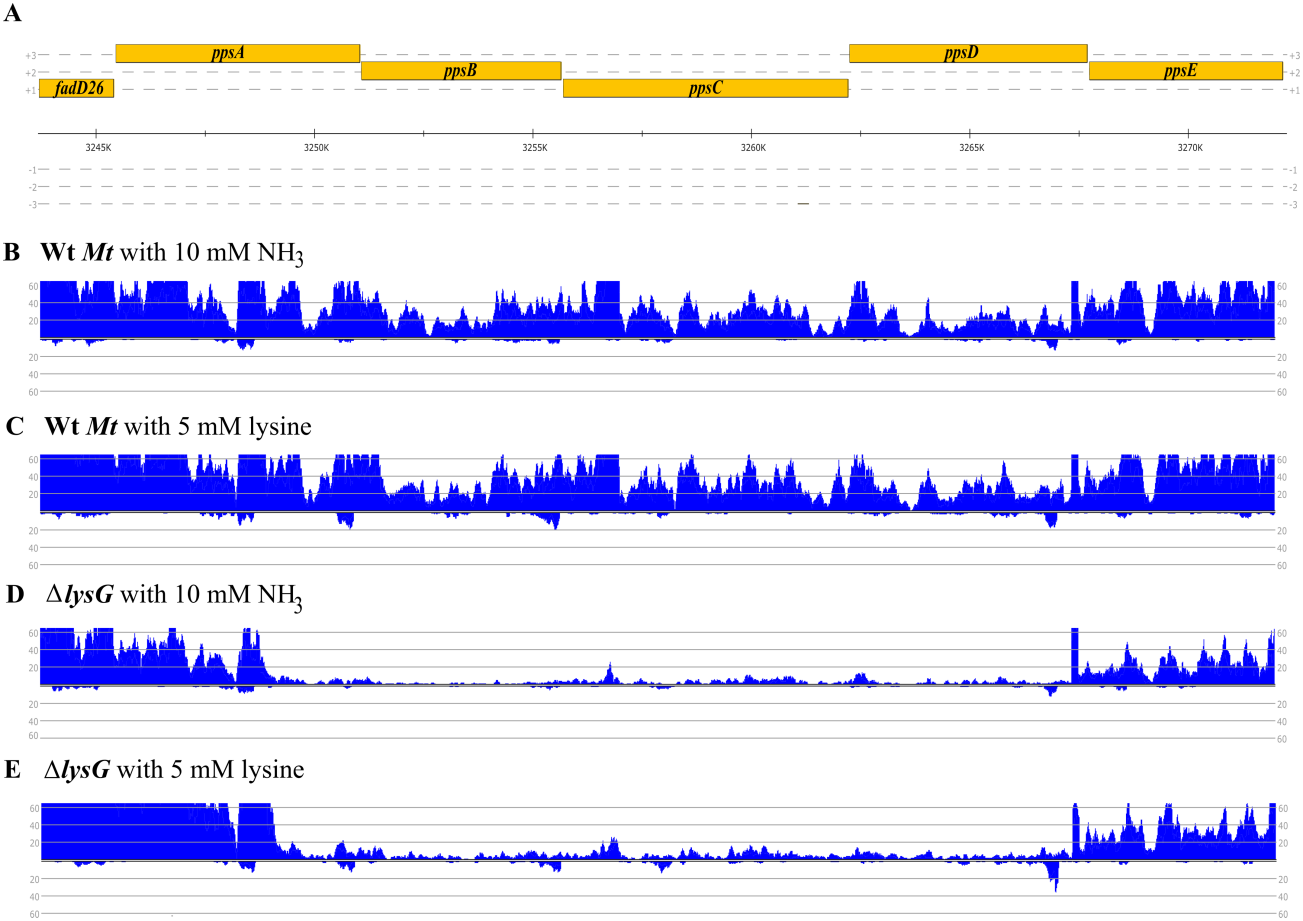
Transcriptome profiles of the DNA region from *fadD26* to *ppsE*. (A) Genomic organization of the genes *fadD26* to *ppsE* of *Mt* is shown. (B) and (C) Profiles of mapped reads from RNAseq using RNA samples from Wt *Mt* with ammonium (B) or lysine (C) as nitrogen source. (D) and (E) Profiles of mapped reads from RNAseq using RNA samples from the Δ*lysG* mutant strain with ammonium (D) or lysine (E) as nitrogen source.

Again, we used data form transcriptome sequencing (RNAseq) to map the promoter and transcription start sites within the *fadD26*-*ppsE*-region. A strong promoter is located in front of *fadD26*. A differential transcription of a region extending from the 3’-prime region of *ppsA* to *ppsD* is seen between the Wt *Mt* and the Δ*lysG* mutant strain (Fig 8). However, in the Wt *Mt* the RNA profile of the *pps*-region does not differ between cultures with ammonium and lysine. The latter is in contrast to the RNA profile of *lysE*-expression, which shows a differential regulation in the Wt *Mt* dependent on lysine. In addition, transcript levels decrease well upstream of the LysG(^Mt^) binding region in front of *ppsB*, as identified by EMSA (Fig 7), and no clear transcription start site in this region is apparent.

## Discussion

LysG(^Mt^) of *Mt* has six characteristics for a classic LTTR: (1) It has an HTH motif at the N-terminus. (2) The peptide length is in the range of other LTTRs. (3) There is an inverted repeat in the center of the promoter region. (4) The intergenic region between *lysG*(^Mt^) and its target gene *lysE*(^Mt^) is short (108 bp). (5) The promoter binding motif of *lysG*(^Mt^) overlap with that of the distal promoter of *lysE*(^Mt^). (6) *LysG*(^Mt^) is divergently transcribed from *lysE*(^Mt^). In *Mt*, this type of genomic organization has further been shown for the transcriptional regulator Rv1404 and Rv3291c, whose genes are directly located up- or downstream of their target genes [39,40]. Earlier studies showed, that the target genes of LTTRs in other bacteria, such as ArgP of *E*. *coli* and LysG of *C*. *glutamicum* are *argO* and *lysE*, respectively [12,13]. ArgO and LysE are amino acid exporters with strong similarities to LysE(^Mt^). Lysine and histidine are co-effectors of LysG-dependent regulation of *lysE* in *C*. *glutamicum* [12]. We showed in *Mt*, that lysine and histidine, are also required for the control of *lysE*(^Mt^) by LysG(^Mt^). Although we did not characterize the function of LysE(^Mt^) in this study, it is probably reasonable to assume that LysE(^Mt^) might also be an amino acid exporter. In *C*. *glutamicum* the gene expression of *lysE* by LysG is activated 20-fold in the presence of lysine and in the presence of histidine 10-fold [11,12]. In *Mt*, activation of *lysE*(^Mt^) is 10-fold for lysine and 4-fold for histidine. Based on a three-dimensional model of LysG(^Mt^), Zhou *et al*. suggested that arginine could also act as a co-effector [21]. This assumption was not confirmed in our experiments. We also excluded other amino acids such as asparagine, aspartate and leucine as co-effectors for LysG(^Mt^)-dependent regulation of *lysE*(^Mt^). Activation of gene expression in the presence of amino acids or vitamins has been demonstrated before in *Mt* for LrpA, a global regulator involved in stress response [40].

We showed that LysG(^Mt^) binds upstream of *lysE*(^Mt^), where a palindromic DNA sequence is located. Palindromic DNA sequences are typical binding regions for regulators, as previously shown for the Rv3334 protein in *Mt* [41]. In general, palindromic DNA sequences are separated by only a few (4 to 5) nucleotides. However, in *Mt* the inverted repeat contains a wider spacer of 11 nucleotides at its center. This indicates that binding at the inverted repeat might cause a DNA bending, which could interfere with transcription. In *S*. typhimurium, it has been shown that, in absence of its co-effector acetyl-_L_-serine, the LTTR CysB binds the *cysK* promoter at two sites [42]. Binding at both sites cause the DNA to bend, and results in inhibition of *cysK* transcription [42]. The authors propose that once acetyl-_L_-serine binds the CysB protein, a conformational change occurs and the CysB protein loses its binding affinity for one binding region. This might cause the DNA curvature to relax and transcription starts [42]. The mechanism might be similar in *Mt*. This idea is supported by our findings that LysG(^Mt^) binds independently of the co-effector lysine and histidine to the promoter region of *lysE*(^Mt^). However, for LysG(^Mt^) to control expression of *lysE*(^Mt^), lysine or histidine are required. Due to divergent promoters, many LTTRs activate expression of their target genes but also repress their own expression [11,41–45]. We suggest that repression of *lysG*(^Mt^) and activation of *lysE*(^Mt^) is possible by using different promoters. Binding of LysG(^Mt^) to the distal promoter (P1-lysE) probably sterically interferes with binding of a sigma factor. At the same time, it facilitates access of RNA polymerase to the proximal promoter (P2-lysE) of *lysE*(^Mt^). Autorepression has already been demonstrated in *Mt*. In a recent study, it has been shown that the Rv3334 protein in *Mt* binds and represses its own promoter, while in absence of the Rv3334 protein the promoter activity was increased [41]. We also found autorepression of *lysG*(^Mt^). Once the regulator gene *lysG*(^Mt^) is deleted, no functional protein is synthesized. Thus, the *lysG*(^Mt^)-expression is no longer inhibited, resulting in an increased expression of *lysG*(^Mt^) for the mutant strain Δ*lysG* compared to the wild type strain.

Furthermore, LysG(^Mt^) influenced transcription of genes involved in PDIM-synthesis [38]. PDIM is a major lipid for cell wall of *Mt* [46]. Synthesis and transport requires a gene cluster of 13 genes (*fadD26* to *mmpL7*) [38]. This gene cluster includes five polyketide synthases, namely PpsABCDE, that are responsible for the synthesis of phthiocerol [38], a major component of PDIM [47]. We showed that three of these, *ppsBCD*, are upregulated by LysG(^Mt^). It is noteworthy that the genes from *fadD26* to *ppsD* overlap unidirectional (four nucleotides) and out-of-frame on the same strand. This indicates a polycistronic mRNA which includes five genes that might be transcribed as a unit, as known for the *lac* operon in *E*. *coli* [48]. It is unusual that only three of five genes, responsible for the synthesis of phthiocerol, are regulated. We demonstrated that LysG(^Mt^) binds upstream of *ppsB* and not upstream of *fadD26*, which is the first gene of a transcription unit of five genes, each which overlap. It is possible that LysG(^Mt^) co-regulates the genes *ppsBCD*, while another transcriptional regulator controls the whole transcription unit (*fadD26* to *ppsD*). Another possibility is a constitutive expression of the genes of the entire gene cluster, while LysG(^Mt^) only upregulates the expression of *ppsBCD* under certain conditions. However, RNAseq data gave no support for a transcription start site upstream of *ppsB* and no evidence, that this activation is dependent on the effector lysine. We also showed that *pps*-transcription is affected upstream of the LysG(^Mt^)-binding site. At present, we have no consistent regulatory model that accommodates the LysG(^Mt^) binding within the *pps* operon and its effect on *pps*-transcription.

Altogether, this study showed that LysG(^Mt^) is a LTTR that activates the gene *lysE*(^Mt^) in the presence of lysine and histidine and downregulates its own transcription. We confirmed that LysG(^Mt^) binds the promoter region of *lysE*(^Mt^) independently of lysine or histidine and found a palindromic DNA sequence upstream from *lysE*(^Mt^), the binding region of LysG(^Mt^). Moreover, we showed that LysG(^Mt^) binds the upstream region of *ppsB* where it exerts a probably indirect and positive effect on transcript amounts of the genes *ppsBCD*.

## Supporting information

S1 Fig PCR and Southern blot analysis to confirm the mutant strain Δ*lys*G. (A) PCR analysis of the Wt *Mt* (lane 1) and the Δ*lys*G mutant strain (lane 2). For DNA- amplification in the upper part, primer flanking the deletion were used (primer lysG fwd and rev, see Table 1), generating a 1.14 kb DNA fragment for the Wt *Mt* and a 0.78 kb DNA fragment for the Δ*lys*G mutant strain. For DNA-amplification in the lower part, primer within the deletion were used (primer Del fwd and rev, see Table 1), generating a 0.44 kb DNA fragment for the Wt *Mt* and no fragment for the Δ*lys*G mutant strain. (B) For Southern blot analysis, genomic DNA was digested with *Ale* I. *Ale* I cuts once within the deletion, leading to an upshift of the 1.0 kb DNA Wt-fragment (lane 1) to a 2.7 kb fragment in the Δ*lys*G mutant strain (lane 3). Lane 2 shows the co-integration of the plasmid used for transformation after the first crossing over, before counter-selection with sucrose was performed.
(TIF)

S2 Fig Sequence analysis of LysE(^Mt^). (A) Complete multiple alignment of three LysE superfamily members demonstrates their homology. Conserved amino acids are indicated in orange and fully conserved in red. (B) The hydropathy plot of LysE(^Mt^) demonstrates an almost identical plot with LysE of *C*. *glutamicum* and indicates five transmembrane-spanning helices for LysE(^Mt^). The average local hydrophobicity at each residue is shown according to the algorithm of Kyte and Doolittle [37], plotted on the vertical axis versus the residue number on the horizontal axis. The sequence of LysE(^Mt^) is matched to the sequence of LysE of *C*. *glutamicum* (multiple alignment). The six bars indicate the location of potential transmembrane-spanning helices.
(TIF)

S1 Table Whole transcriptome expression analysis. Wt *Mt* and Δ*lysG* were grown in minimal medium in the presence of 5 mM lysine as sole nitrogen source. Signal intensities of the mutant strain Δ*lysG* were compared to the Wt *Mt* strain. A dataframe with a row for the number of top genes is presented. Fold changes (FC) were expressed as mean FC from three independent experiments (Exp).
(XLSX)
