# Supplementary figures and images for: The transcriptional regulator LysG (Rv1985c) of Mycobacterium tuberculosis activates lysE (Rv1986) in a lysine-dependent manner
Source: PLoS One. 2017 Oct 19;12(10):e0186505. doi: 10.1371/journal.pone.0186505 (PMC5648196; doi:10.1371/journal.pone.0186505)

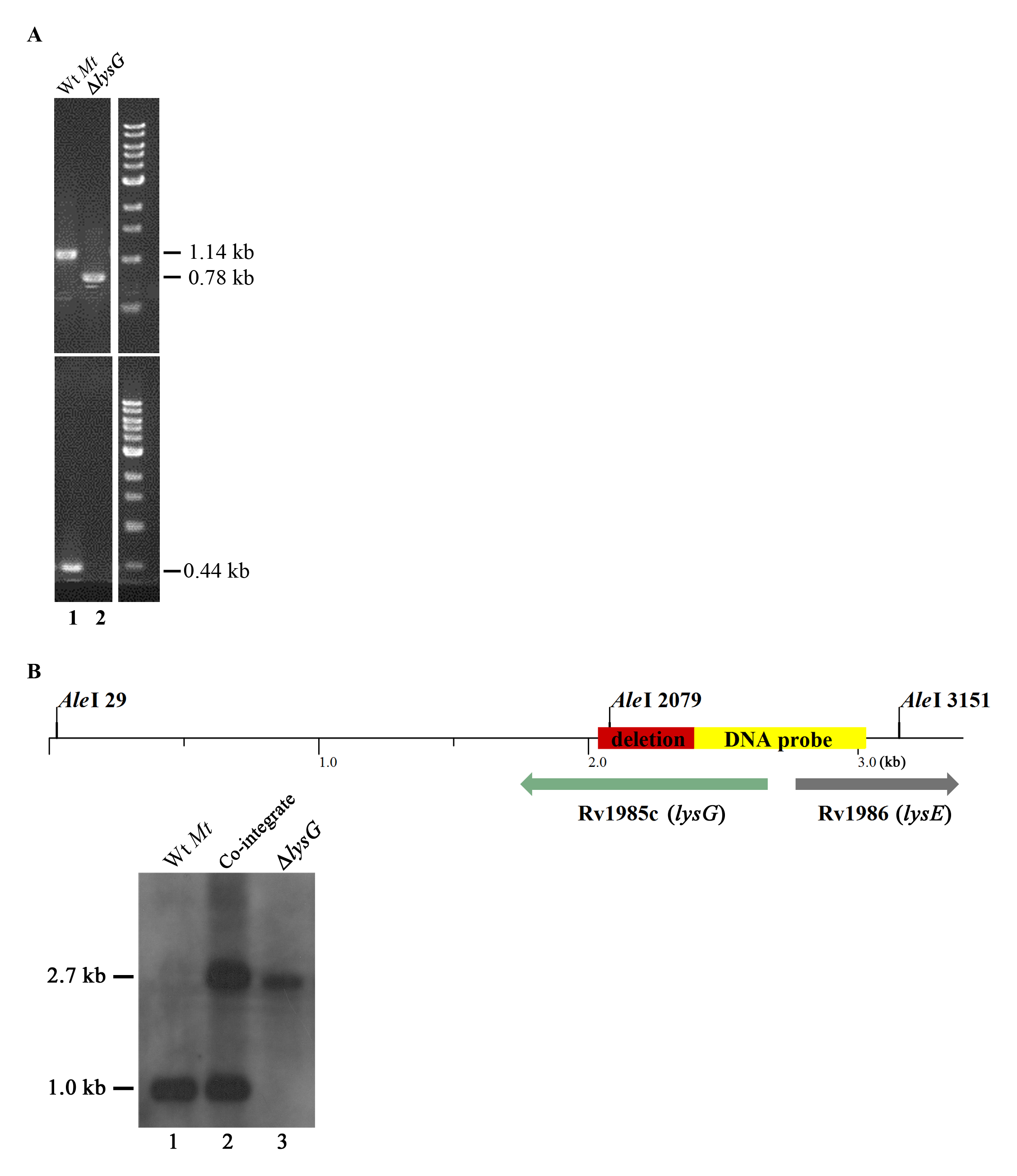

Supplement: S1 Fig — (A) PCR analysis of the Wt Mt (lane 1) and the ΔlysG mutant strain (lane 2). For DNA- amplification in the upper part, primer flanking the deletion were used (primer lysG fwd and rev, see Table 1), generating a 1.14 kb DNA fragment for the Wt Mt and a 0.78 kb DNA fragment for the ΔlysG mutant strain. For DNA-amplification in the lower part, primer within the deletion were used (primer Del fwd and rev, see Table 1), generating a 0.44 kb DNA fragment for the Wt Mt and no fragment for the ΔlysG mutant strain. (B) For Southern blot analysis, genomic DNA was digested with Ale I. Ale I cuts once within the deletion, leading to an upshift of the 1.0 kb DNA Wt-fragment (lane 1) to a 2.7 kb fragment in the ΔlysG mutant strain (lane 3). Lane 2 shows the co-integration of the plasmid used for transformation after the first crossing over, before counter-selection with sucrose was performed. (TIF) [file pone.0186505.s001.tif]

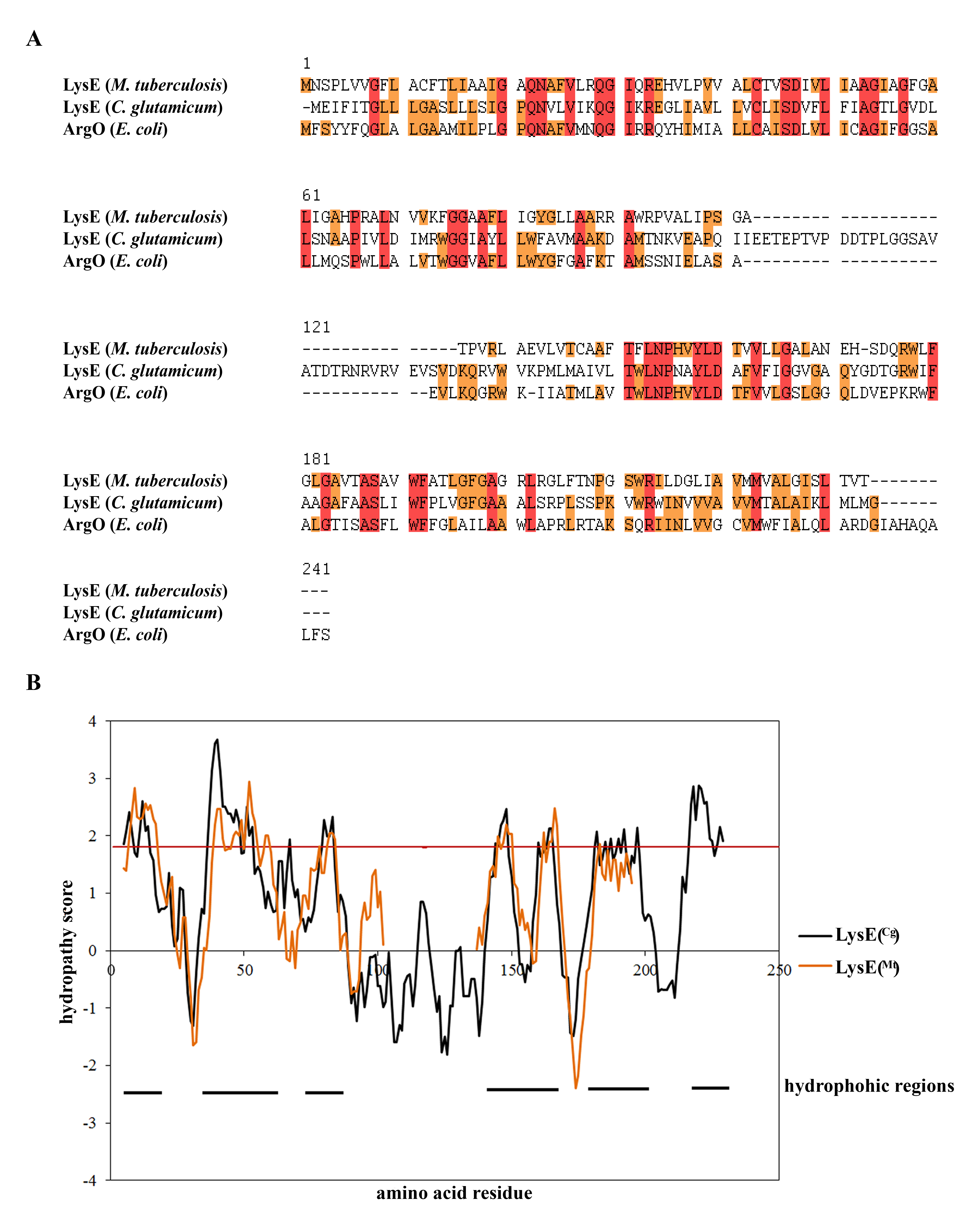

Supplement: S2 Fig — (A) Complete multiple alignment of three LysE superfamily members demonstrates their homology. Conserved amino acids are indicated in orange and fully conserved in red. (B) The hydropathy plot of LysE(Mt) demonstrates an almost identical plot with LysE of C. glutamicum and indicates five transmembrane-spanning helices for LysE(Mt). The average local hydrophobicity at each residue is shown according to the algorithm of Kyte and Doolittle [37], plotted on the vertical axis versus the residue number on the horizontal axis. The sequence of LysE(Mt) is matched to the sequence of LysE of C. glutamicum (multiple alignment). The six bars indicate the location of potential transmembrane-spanning helices. (TIF) [file pone.0186505.s002.tif]
